# Improved A-Star Search Algorithm for Probabilistic Air Pollution Detection Using UAVs

**DOI:** 10.3390/s24041141

**Published:** 2024-02-09

**Authors:** Il-kyu Ha

**Affiliations:** School of Computer Science, Kyungil University, Gyeongsan 38428, Republic of Korea; ikha@kiu.kr; Tel.: +82-53-600-5564

**Keywords:** unmanned aerial vehicles, probabilistic search, air pollution detection, UAV air pollution detection

## Abstract

Recently, air pollution problems in urban areas have become serious, and unmanned aerial vehicles (UAVs) can be used to monitor air pollution because they can perform spatial movement. However, because air pollution sources are fluid, probabilistic search methods are required to identify a target through the probability of its existence. This study proposes an efficient algorithm to detect air pollution in urban areas using UAVs. An improved A-star algorithm that can efficiently perform searches based on a probabilistic search model using a UAV is designed. In particular, in the proposed improved A-star algorithm, several special weights are used to calculate the probability of target existence. For example, a heuristic weight based on the expected target, a weight based on data collected from the drone sensor, and a weight based on the prior information of obstacles presence are determined. The method and procedure for applying the proposed algorithm to the stochastic search environment of a drone are described. Finally, the superiority of the proposed improved A-star algorithm is demonstrated by comparing it with existing stochastic search algorithms through various practical simulations. The proposed method exhibited more than 45% better performance in terms of successful search rounds compared with existing methods.

## 1. Introduction

Recently, unmanned aerial vehicles (UAVs) have been used in various fields. In particular, they are being widely used in the field of environmental surveillance owing to their various advantages, including movement in space. UAVs can also be used to explore polluted areas on the ground, discover various types of pollutants in rivers or the sea, and identify atmospheric pollutants such as NO, CO, CO_2_, and fine dust. Therefore, when a UAV searches for various targets on the ground or in the air, it may not be a good idea for a person to directly operate the UAV and search for the target. This is due to the time constraints for target discovery, restrictions on quick decisions, and actions for target judgment. Therefore, environmental exploration using UAVs requires a method that enables a UAV to perform its mission according to a preplanned autonomous flight algorithm [1]. When a UAV is used to detect targets on the ground or in space, autonomous flight methods that can be applied to the UAV include navigating a preplanned path and searching for the target by relying on the information collected while the drone is flying. In the first method, “the method of exploring a preplanned path [2]”, the target’s location is estimated in advance before flight, the path is input to the UAV, and the UAV navigates accordingly. The second method, “the method of searching for a target based on information collected while flying [3]”, uses data such as images, videos, sounds, signals, and smoke collected while flying to determine the target, and the UAV determines the path accordingly. This method can be considered to have relatively higher usability than the first method. Data such as images, videos, sounds, signals, and smoke collected by UAVs through sensors are used to identify the presence of a target using a target-determination algorithm. In air pollution exploration using UAVs, the collected data are likely to be images or gases such as smoke. An appropriate target-determination algorithm is necessary to determine the existence of a target based on various types of collected data. When determining a target using data such as collected images and videos, the target can be determined by comparison with a previously predicted target image. To achieve this, the probability of target judgment can be increased through artificial intelligence methods, such as machine learning or deep learning. When judging a target using collected sounds, signals, and smoke, the target can be identified by determining the strength and accuracy of the signal [4,5]. To determine the source of air pollution using UAVs, various sources of pollution can be identified through sensors, and the presence and area of occurrence of the source can be determined based on their intensity and accuracy. However, as air pollution sources are both fluid and invisible, finding air pollution through sensors is difficult for drones. In such cases, a probabilistic search method that finds the place with the highest presence probability of air pollution can be used as an alternative. The probabilistic target search method is used to identify a target by increasing the probability of its existence based on data collected through repeated searches and a probability model [6,7,8,9,10].

This study focuses on improving the probabilistic search method used to identify air pollution in urban areas using UAVs. The A-star algorithm is a traditional method for finding the shortest path in space. The improvement of the A-star algorithm to improve the mobility of drones in air pollution detection was studied, and an improved A-star model with additional parameters suitable for air pollution detection was proposed. The proposed model introduces new parameters such as heuristic weights, weights measured by sensors, and weights for obstacles to improve the navigation performance. This improved model is applied to several comparable existing stochastic search methods, and their performance is compared. In other words, the performance difference is analyzed by comparing cases where the improved A-star model is applied and cases where it is not applied.

Therefore, this study has the following contributions:

- First, this study presents an improved A-star model that can improve search performance when using a stochastic model to search for air pollution in urban areas. To improve air pollution detection performance, appropriate parameters are introduced and applied to the improved A-star algorithm. In particular, heuristic estimates for targets, weights for obstacles, and weights determined by drone sensors are considered.
- Second, an idea is presented to improve the search performance of a probabilistic model based on Bayes’ theorem. To date, in several studies, a probabilistic search model based on Bayes’ theorem has been presented to improve the performance of drone search. This study can improve the performance of the Bayes’-theorem-based search model.
- Third, ideas on the use of drones to detect air pollution in urban areas are presented. Ideas about various uses of drones have been presented in several studies, which will be discussed in the next section. However, relatively few ideas about exploring air pollution have been discussed. Therefore, because the interest in environmental pollution is increasing, this study can mitigate the environmental problems using UAVs.

## 2. Related Work

The section summarizes the research and methods employed for target searches using UAVs. The section is appropriately divided into two sub-sections: UAV navigation-related studies and existing probabilistic-model-based search algorithm.

### 2.1. UAV Navigation-Related Studies

Several studies have used UAVs to search for targets. Such cases can be classified according to the criteria listed in Table 1.

The flight operation method can be divided into route planning and autonomous flights. In the route planning method, the optimal route is planned, and the drone searches according to the planned route. Autonomous flight is a method in which a UAV performs its mission according to a preplanned algorithm. There are studies [2,11,12] related to route planning methods. Perazzo et al. [2] studied how drones fly through waypoints instead of fixed anchors on the ground and how to determine and navigate efficient flight paths. Hayat et al. [11] addressed the dynamic path-planning problem of UAVs for search and rescue missions. Shivgan et al. [12] studied a method for optimizing the flight path using the traveling salesman algorithm, considering the limited UAV flight time. Yao et al. [13] studied the most appropriate route planning when attempting to carry out search missions in river areas using UAVs. The goal of this route planning is to create a route with the maximum discovery probability for a single fixed target, taking into account the importance of the distribution of regions. Chen et al. [14] studied an ant colony system (ACS)-based heuristic algorithm that allows heterogeneous UAVs to efficiently and fully explore the coverage. The algorithm consists of an area allocation step and an order optimization step, and pheromones and heuristic information derived from ACS parameter values are used for optimization. Chen et al. [15] studied the problem of cooperatively controlling the behavior of UAVs in a global or local area. To solve this problem, they presented a reinforcement learning-based approach to derive dynamic action sequences of UAVs and recognize cooperation. The route planning method has the disadvantage of not being able to properly adjust the route according to environmental changes because it searches a predetermined route according to an algorithm. However, because it searches for a set route, it has the advantage of reducing unnecessary search distance and time. Autonomous flight methods are used in target searches with most UAVs. These include [3,16,17,18,19]. Chuang et al. [3] addressed the problem of autonomous target estimation by using drones. They proposed an autonomous UAV control method using high-performance cameras. Wu et al. [16] studied a method for finding a flying target path through in-depth intensive learning and training for the autonomous control of drones. Deng et al. [17] studied the manner in which a UAV equipped with a vision system for surveillance applications autonomously detected and tracked target objects without human intervention. Rabah et al. [18] studied a control method for a quadcopter that tracked a moving target. Cheng et al. [19] proposed a method for detecting small UAV targets to maintain security in urban areas. Autonomous flight methods have the advantage of being able to optimize navigation by appropriately adjusting the route according to changes in the navigation environment. However, it has the disadvantage that the distance and time to perform the mission may increase depending on environmental changes.

Second, in addition to target tracking and judgment methods, probability-based and artificial intelligence methods have been proposed. As mentioned previously, the probability-based method calculates the degree of target matching as a probability based on the data collected by the UAV, and a probabilistic model increases the probability through an iterative method and judges it as a target when it reaches a certain probability value. Artificial intelligence methods have been widely studied recently. These methods determine the existence of a target by determining whether it matches the image of the target planned in advance, based on the image and video data collected by the UAV. Recently, various machine and deep learning methods have been used to increase the probability of target recognition. Research on probabilistic searches includes [20,21,22,23,24,25,26,27], and search methods using artificial intelligence include [28,29,30,31,32]. Symington et al. [20] conducted an early study on stochastic searches. They proposed a target detection algorithm based on a recursive Bayesian model that estimated the probability of a target being present in video frames collected from a camera mounted on a UAV. Serna et al. [21] focused on improving the performance of UAVs used for planetary exploration. They presented a high-level probabilistic search method for planetary exploration, based on the Partially Observable Markov Decision Process. Naula et al. [22] proposed an algorithm for detecting pollutants in the air using a UAV. The proposed algorithm uses both metaheuristic and probabilistic search methods. Chung [23] expressed the search problem in a probabilistic formulation that can be used when a search agent, such as a UAV, finds a fixed target in the search area. Carrese et al. [24] presented a method of using a UAV to determine the location of wild-parked e-scooters in urban areas, and they proposed an efficient heuristic model for this purpose. Mozaffari et al. [25] studied the appropriate altitude and coverage for UAVs to function as wireless base stations. They derived the coverage probability of a UAV using altitude and antenna gain functions and studied how to maximize the coverage of a UAV. Trotta et al. [26] studied how to efficiently perform video monitoring in urban areas using UAVs that move to search points while charging on a bus. In particular, they use a probabilistic model to calculate the efficient navigation coverage of UAVs. Alawad et al. [27] presented a system for collecting and processing disaster information by deploying a group of UAVs at disaster sites in urban areas. In particular, they used a probabilistic model to calculate the energy-efficient navigation coverage of the drone. The probabilistic search method repeatedly searches the search space and increases the probability of success in the search; therefore, it has the disadvantage that the search distance and time can be long. However, this method can increase the likelihood of search success when the search target is not clear. Most artificial intelligence search methods use YOLO (You Only Look Once) deep learning technology. The YOLO model introduced by Redmon et al. in 2015 [33] detects objects immediately after looking at the image only once. Tan et al. [28] and Luo et al. [29] proposed an improved algorithm using the existing YOLO v4 algorithm to improve the target detection performance from images captured by UAVs. Luo et al. [30] proposed an improved YOLO v5 algorithm using the k-means++ algorithm. Zhu et al. [31] focused on improving the recognition accuracy of fruit tree canopies in orchards captured by UAVs and proposed an improved YOLO v4. Wang et al. [32] proposed an online distributed algorithm for tracking and searching for object detection and search trajectory planning for the security surveillance of UAVs with relative mobility and scalability. They also proposed a quantum probability model that partially explains the observable target location. In artificial intelligence search methods, environmental data must be collected, processed, and applied to search activities in real time; therefore, the data processing cost of search drones is high. However, search drones can perform the most appropriate activities depending on environmental changes.

Third, studies can be categorized based on the data collected to determine the target. The types of data collected included images, videos, sounds, smoke, and signals. Images and videos can be used to detect specific types of targets on the ground. Refs. [34,35,36,37] are related to this topic. Minaeian et al. [34] presented an algorithm for efficiently determining and tracking the location and movement of crowds in images acquired by UAVs in border areas. Liu et al. [35] proposed a multitarget tracking algorithm based on YOLO v4 to identify and track vehicles in urban areas using UAVs. Ren et al. [36] proposed a Mask-R-CNN algorithm that can efficiently process video streams acquired by UAVs in terms of speed and storage space. Mandal et al. [37] proposed an image dataset that efficiently recognizes moving objects in video images collected by UAVs. In addition, several studies have demonstrated that UAVs can detect objects using sounds produced by the target [38,39]. Zimroz et al. [38] studied the use of UAVs for search and rescue activities in underground mines. They proposed a method for detecting specific sounds from acoustic data mixed with noise obtained from underground passages. Yang et al. [39] studied methods for detecting and determining the path of a flying UAV using sounds generated by the UAV. The UAV detects the signals generated from the target, tracks the target, and collects data; related studies include [40,41]. Ebrahimi et al. [40] addressed the efficient collection of data using UAVs in wireless sensor networks. The UAV detects the signals generated by the sensor nodes and collects data. Abro et al. [41] proposed a method for identifying and detecting illegal UAVs in urban areas using signals generated by a UAV Controller Device. Smoke is gaseous data that can be collected by a UAV, including data on various air pollutants such as CO, CO_2_, NO, NO_2_, and fine dust distributed in the air. Studies related to this topic have been conducted [3,9,42]. Yuan et al. [42] used UAVs to identify missing people or objects in remote wilderness areas. They proposed a method for locating and tracking a target based on gases emitted from the target. Lambey et al. [9] studied the types of UAV sensors used for air quality monitoring based on an extensive literature review. Pochwala et al. [43] proposed a UAV-based air pollution measurement system that can detect harmful compounds, such as ammonia, hexane, benzene, and CO, and combustible substances, such as hydrogen and methane, in the atmosphere. Methods for searching images, videos, sounds, signals, smoke, etc., use an optimal sensor suited to the purpose of the search. Therefore, data suitable for exploration purposes can be appropriately collected, but other data cannot be collected.

UAVs are suitable for exploring air pollution in urban environments. This is because they exhibit superior mobility compared with other air pollution measurement devices. However, because air pollution possesses dynamic characteristics, a search algorithm that takes these into account is necessary.

This study focused on gas data and algorithms that can search for sources of air pollution. Therefore, this study can be classified as follows: autonomous flight as the flight manipulation method, probability-based method as the target tracking and judgment method, and method for collecting smoke data as the collected data.

The method proposed in this study is classified as an autonomous flight method. This means that the method can be applied as an autonomous flight method for actual drones. Additionally, this method is classified as a stochastic search method. The target of drone exploration in this study is air pollution. Air pollution is not visible and fluctuates depending on environmental changes; therefore, it is very difficult to detect with drone sensors. Therefore, owing to the fluid nature of air pollution, probabilistic search is the subject of this study to increase the likelihood of successful search, and an improved A-star algorithm is proposed to efficiently execute this probabilistic search. Therefore, one of the unique features of this study is the application of the improved A-star algorithm suitable for air pollution to the stochastic search method.

**Table 1:** UAV navigation-related studies classified into major categories.

Category	Type	Study
Flight control	Route planning	[2,11,12,13,14,15]
	Autonomous flight	[3,16,17,18,19]
Target tracking and judgment	Probabilistic search	[20,21,22,23,24,25,26,27]
	Artificial intelligence	[28,29,30,31,32]
Collection data	Image	[34,35]
	Video	[36,37]
	Sound	[38,39]
	Signal	[40,41]
	Smoke	[7,42,43]

### 2.2. Probabilistic-Model-Based Search Algorithm

The probability-based search model proposed by Chung [6] (Equation (1)) is currently being explored and utilized in many studies. This model is expressed as the probability of a false alarm (*α*) and missed detection (*β*), depending on whether an actual target exists in the search area and the result of the UAV search. In this equation, $d_{a}^{t}$ denotes the value of determining whether a target exists in cell ‘a’ at time *t*, and $x_{T}=a$ indicates that a target exists in search area ‘a’. This equation expresses the probability of an error occurring as *α* and *β* when the drone identifies the target. *α* and *β* stand for false alarm and missed detection, respectively. For example, $Pr\left(d_{a}^{t}=0|x_{T}=a\right)$ is the probability determined as $d_{a}^{t}=0$ under the condition $x_{T}=a$. In other words, the condition is that there is a target in search area a, but this means that the drone failed to search for the target, thus indicating a missed detection.

(1) $$\mathrm{Pr}\left(d_{a}^{t}|x_{T}\right):\;\left\{\begin{array}{c} Pr\left(d_{a}^{t}=0|x_{T}=a\right)=\beta ,\\ Pr\left(d_{a}^{t}=1|x_{T}=a\right)=1-\beta ,\\ Pr\left(d_{a}^{t}=0|x_{T}\neq a\right)=1-\alpha ,\\ Pr\left(d_{a}^{t}=1|x_{T}\neq a\right)=\alpha , \end{array}\right.$$

If $d^{t}\;$ is the *t*-th observation, $D^{t}$ is the set of *t*-th observations, and $x_{T}=1$ indicates that a target exists in a specific area, then the probability of the target at time *t* is calculated using Bayes’ rule [44] as follows [20]:

(2) $$P_{r}\left(x_{T}=1|D^{t}\right)=\frac{P_{r}\left(d^{t}\;\right|x_{T}=1)\;P_{r}(x_{T}=1|D^{t-1})}{P_{r}\left(d^{t}\;\right|D^{t-1})}$$

Equations (3) and (4) based on Bayes’ rule express the relationship between the prior and posterior probabilities of two random variables.

(3) $$P\left(A|B\right)=\frac{P\left(B|A\right)\cdot P(A)}{P(B)}$$

(4) $$P\left(A|B\right)=\frac{P\left(B|A\right)\cdot P(A)}{P\left(B|A\right)\cdot \;P\left(A\right)+P(B|\neg A)\cdot P(\neg A)}$$

If Equation (2) is expressed in the same form as Bayes’ rule (Equation (4)), it can be expressed as follows:

(5) $$P_{r}\left(x_{T}=1|D^{t}\right)=\frac{P_{r}\left(d^{t}\;\right|x_{T}=1)\;P_{r}(x_{T}=1|D^{t-1})}{P_{r}\left(d^{t}\;\right|x_{T}=1)\;P_{r}\left(x_{T}=1|D^{t-1}\right)+P_{r}\left(d^{t}\;\right|x_{T}\neq 1)\;P_{r}(x_{T}\neq 1|D^{t-1})\;}$$

In the above equation, if $d^{t}=1$, that is, if it is determined that there is a target, applying the Bayesian rule to Equation (5) is expressed as Equation (6).

(6) $$\frac{\left(1-\beta_{h}\right)P_{t-1}}{\left(1-\beta_{h}\right)P_{t-1}+\alpha_{h}(1-P_{t-1})},\;\;\;\;\;\;if\;d^{t}=1$$

In Equation (6), because $d^{t}$ is judged to be 1 under the condition $x_{T}=1$, the coefficient of $P_{t-1}$ is $1-\beta_{h}$, and because the probability of $d^{t}$ is judged to be 1 under the condition $x_{T}\neq 1$, the coefficient of $1-P_{t-1}$ becomes $\alpha_{h}$ (false alarm).

If $d^{t}$ is 0 in the above equation, that is, if it is determined that there is no target, by applying the Bayes rule to Equation (4), Equation (7) is obtained as follows:

(7) $$\frac{\left(\beta_{h}\right)P_{t-1}}{\left(\beta_{h}\right)P_{t-1}+(1-\alpha_{h})(1-P_{t-1})},\;\;\;\;\;\;if\;d^{t}=0$$

In Equation (7), because $d^{t}$ is judged to be 0 under the condition $x_{T}\neq 1$, the coefficient of $1-P_{t-1}$ is $1-\alpha_{h}$, and because the probability of $d^{t}$ is judged to be 0 under the condition of $x_{T}=1$, the coefficient of $P_{t-1}$ becomes $\beta_{h}$ (missed detection).

Therefore, by combining both equations, Equation (8) is derived. This equation can be used in circular and iterative probabilistic searches because the next probability $P_{t}$ is obtained using the value of $d^{t}$ repeatedly determined with the value of $P_{t-1}$ [6,20].

(8) $$P_{t}=\left\{\begin{array}{c} \frac{\left(1-\beta_{h}\right)P_{t-1}}{\left(1-\beta_{h}\right)P_{t-1}+\alpha_{h}(1-P_{t-1})},\;\;\;\;\;\;if\;d^{t}=1\\ \frac{\left(\beta_{h}\right)P_{t-1}}{\left(\beta_{h}\right)P_{t-1}+(1-\alpha_{h})(1-P_{t-1})},\;\;\;\;\;\;if\;d^{t}=0 \end{array}\right.$$

In other words, the probability $P_{t}$ that the target exists in a certain cell in the search space can be cyclically obtained using the previous search probability $P_{t-1}$, the probability $\alpha_{h}$ of a false alarm at altitude h, and the probability $\beta_{h}$ of missed detection at altitude h.

## 3. Improved A-Star Algorithm

This section describes an improved A-star algorithm for probabilistic air pollution detection using a UAV. The conventional A-star algorithm is improved to detect air pollution in urban areas using UAVs, and the improved algorithm is applied to the probabilistic search model. The process of improving the conventional A-star algorithm to be suitable for air pollution search by adding search distance and direction information and obstacle information is explained. Additionally, a method for applying the improved model to a probabilistic search model is presented.

### Improved A-Star (A*) Search Algorithm

The conventional A-star algorithm searches for the shortest path in space and determines it based on heuristic estimates and weight values for motion. This algorithm is expressed as Equation (9), where $G\left(n\right)$ is the weight of the path from the starting point to the current point, and $H\left(n\right)$ is the weight of the estimated path from the current point to the target point. In general, $H\left(n\right)$ is obtained by estimating the distance from the current point to the target point while ignoring obstacles in space, and $G\left(n\right)$ is obtained by accumulating the path weights from the starting point to the current point [45,46].

(9) $$F\left(n\right)=G\left(n\right)+H\left(n\right)$$

The basic A-star algorithm can be applied to a model in which a drone searches for a target. The conventional A-star algorithm is used to optimize movement in space. This algorithm includes a directional component toward the target derived from $H\left(n\right)$ and $S\left(n\right)$. However, because the existing probabilistic search model does not have a directional element, an improved A-star algorithm with added elements suitable for air pollution search is proposed and then applied to the probabilistic search model. Therefore, the key features of performance improvement are directional information and obstacle information.

This study proposes an adaptive A-star algorithm that can improve the performance of the stochastic search model. Moreover, this study proposes a method to reduce the search time and distance in the stochastic model and increase the probability of successful target search. The proposed algorithm is expressed as Equation (10). This equation is a combination of Equation (8), which calculates the basic probability of a cell, and the improved A-star algorithm. In the proposed equation, $P\left(n\right)$ inherits the path weight $G\left(n\right)$ from the traditional A-star equation, where $P\left(n\right)$ is the cumulative predicted value, based on the data predicted by the drone in the previous step. $H\left(n\right)$ denotes the estimated distance from the target to the current point. Because efficient search pursues a shorter distance, $H\left(n\right)$ becomes an important factor in determining the direction of search. $O\left(n\right)$ expresses information about obstacles in the search space as weights. Areas where obstacles exist can be assigned a low weight value because the probability of target discovery is low. Most areas without obstacles can be assigned normal weights. In this equation, S(n) is used to increase the probability of search success. $S\left(n\right)$ is also one of the features of the proposed algorithm that improves search performance. This refers to air pollution measurements collected by sensors in the drone’s actual navigation environment. This value becomes an important factor for calculating the search probability, as shown in Equation (10). Therefore, the higher the measurement value, the higher the weight and probability of discovery. Therefore, the prediction function $PF\left(n\right)$ can be expressed as the sum of the previous-step prediction function $P\left(n\right)$, target point distance estimation function $H\left(n\right)$, obstacle weight function $O\left(n\right)$, and sensor data collection weight function S(n).

(10) $$PF\left(n\right)=P\left(n\right)+H\left(n\right)+O\left(n\right)+S(n)$$

The proposed equation was applied to a two-dimensional search space in which the drone searched. It is assumed that the search space consists of several cells with x- and y-axes coordinates. The predicted value at time *t* for a specific cell $c\left(i,j\right)$ can be expressed as Equation (11). The prediction function $P_{t}$ at time *t* can be obtained as the sum of the prediction value $P_{t-1}$ at the previous time *t*−1, the target point distance estimate $H_{t-1}$, the obstacle weight value O, and the sensor-collected data weight value $S_{t-1}$. The obstacle weight value is an element with a constant value that does not change with time and can only be considered once during the initial search; therefore, the time is not displayed.

(11) $$P_{t}\left(i,j\right)=P_{t-1}\left(i,j\right)+H_{t-1}\left(i,j\right)+O\left(i,\;j\right)+S_{t-1}\left(i,j\right)$$

If time *t* is 1, that is, if it is an initial search, Equation (11) can be expressed as Equation (12). If time *t* is greater than 1, repeated searches after the initial search are performed through a probabilistic search using a prediction function, such as Equation (13).

(12) $$P_{1}\left(i,j\right)=P_{0}\left(i,j\right)+H_{0}\left(i,j\right)+{O\left(i,j\right)+S}_{0}\left(i,j\right)$$

(13) $$P_{t}\left(i,j\right)=P_{t-1}\left(i,j\right)+H_{t-1}\left(i,j\right)+S_{t-1}\left(i,j\right)$$

The method by which a drone determines the probability of the existence of a target in each cell in the probabilistic search space uses Equations (12) and (13). The initial probability of the target existence in each cell in the search space is calculated using Equation (12). In addition, the probability of target existence in each cell in repeated searches after the initial search was calculated using Equation (13). In Equation (12), which calculates the initial target presence probability, $S_{0}\left(i,j\right)$ is the initial sensor measurement value given to each cell. $H_{0}\left(i,j\right)$ is the distance between the initial target and the cell assigned to each cell. $O\left(i,j\right)$ is a weight value based on the initial obstacle presence information given to each cell and is a constant value that does not change. This weight is a factor that the drone can consider once at the beginning of the search; therefore, it is included in the initial search but excluded from subsequent repeated searches. In the iterative probabilistic search after the initial search, the probability of the target existence in each cell is calculated using Equation (13). $P_{t-1}$ was calculated using Equation (5). The $S_{t-1}$ value refers to the value measured by the drone’s air pollution source search sensor from the previous step. $H_{t-1}$ is the value estimated based on the distance from each cell to the cell with the highest probability (the expected target point) in the previous step. Therefore, the $P_{t}$ value calculated by reflecting the target point distance estimate value $H_{t-1}$ and the sensor-collected data weight value $S_{t-1}$ is cyclically reflected in the next step of the calculation.

**Figure 1:**
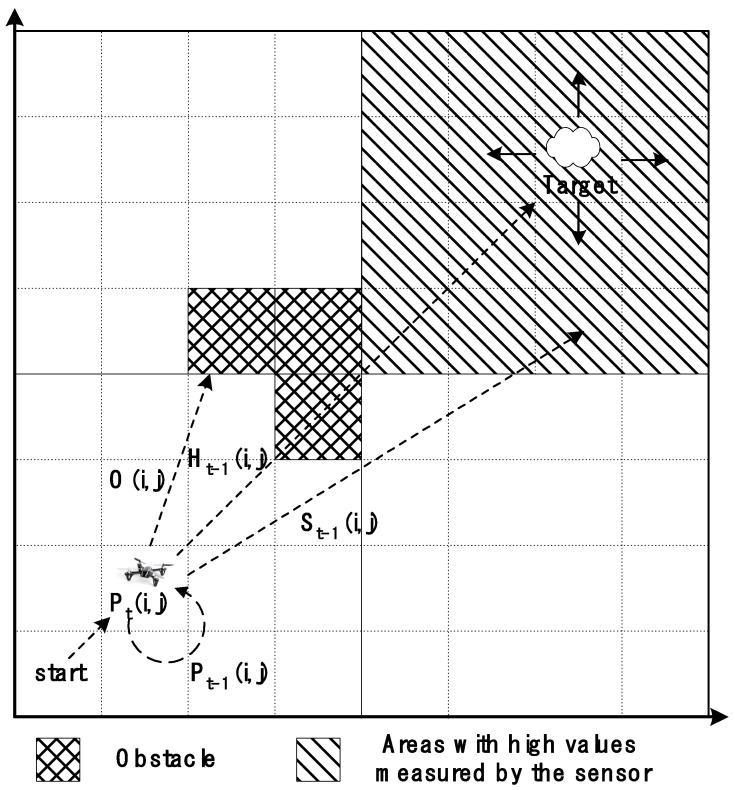
Method for determining the search success probability $P_{t}\left(i,j\right)$ of cell c(i,j) in the air pollution source search space.

In Figure 1, the area marked with the diamond grid pattern represents a cell with an obstacle; the cells included in this area have low $O\left(n\right)$ weight values. The area marked with the diagonal stripe patterned is wide, including the target cell, and represents the area with the highest sensor measurement value when a drone searches for sources of air pollution. In a large-scale probabilistic search, it is extremely difficult to determine the narrow area with the highest number of sensor measurements. Therefore, a reasonable sensor measurement value that could be assigned to each cell in the search area was required. For example, the quadrant containing the cell with the highest target presence probability value may be assumed to be an area with a high sensor measurement value, and a high sensor measurement value may be assigned to that area. In an actual drone air pollution source search, the S(n) value can be determined based on the value detected by the air pollution sensor; however, in the simulation, experimentally determined reasonable weights can be used.

$H\left(n\right)$ and S(n) can be considered the most important factors in the proposed Equation (10) for stochastic target search. First, the calculation of $H\left(n\right)$ in the search area is explained. The cell $C_{t}$ with the highest probability among all cells (*Prob_t_*) in the search area is determined, as given in Equation (14), and the heuristic function (*Heu*) is applied to all cells based on the probability value of cell $C_{t}$ to calculate the heuristic value $H_{t}\left(i,j\right)$ of each cell using Equation (15). In other words, $H_{t}\left(i,j\right)$ is the distance between each cell and the cell with the highest probability $C_{t}$. This distance is obtained as a straight-line distance depending on two- or three-dimensional space. The $Heu(C_{t})$ function calculates the distance value for each cell.

(14) $$C_{t}=\max\;({Prob}_{t})$$

(15) $$H_{t}\left(i,j\right)=Heu(C_{t})$$

The sensor-collected data weight value, $S_{t-1}$, for each cell is obtained as follows: In actual navigation, it is determined by the value detected by the drone’s air pollution sensor. Although the value is easy to measure in the drone’s actual navigation environment, expressing the value in the algorithm is difficult, In other words, it is not appropriate to simply substitute a random value into that value. Therefore, methods and processes to express the value more appropriately were explained as follows. In this study, reasonable weights were obtained using the following method: First, the average value ${MA}_{t}$ for all cells in the search area at time *t* is obtained, as shown in Equation (16). In this equation, height and width denote the number of cells horizontally and vertically. The search area was divided into four quadrants, and the quadrant with the highest $P_{t}$ value was identified. If a specific cell is in the quadrant containing the highest $P_{t}$ value, a high weight value can be obtained, as shown in Equation (17). ${Quad}_{t}$ denotes the four quadrants of the search area. The $H_{t}$ and $S_{t}$ values obtained in this manner were used to calculate the P value, gradually increasing the probability of discovery.

(16) $${MA}_{t}=\sum_{i=1}^{height}\sum_{j=1}^{width}{Prod}_{t}(i,j)/height\;*\;width\;\;$$

(17) $$S_{t}\left(i,j\right)=W\left({MA}_{t}\right)\;if\;c\left(i,j\right)\in \max\left({Quad}_{t}\right)$$

**Figure 2:**
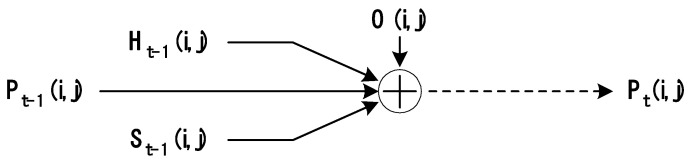
Factors determining the probability of search success for each cell at time *t*.

Figure 2 graphically shows how $P_{t}$ was determined from the $P_{t-1}$.

Based on the equations proposed above, the algorithm for a scenario in which a drone searches for air pollution sources in the search space is presented in Algorithm 1. First, the search prediction value P, target point distance estimate H, obstacle weight O, and sensor-collected data weight S for all the cells are initialized. The flag variable targetFound and the number of search rounds for target discovery were initialized. The search routine was repeatedly executed until the target was found. The predicted value for each cell is obtained from the number of rounds. This was obtained by adding the predicted value from the previous step, the target point distance estimate, and the sensor-collected data weight. Subsequently, the search prediction value for each cell was obtained using the probability model, and it was verified whether the value reached the probability set as the threshold. If the predicted value is above the threshold, the discovery is considered successful, and the value of the targetFound flag variable is changed to 1. If the threshold was not reached, the next round was repeated.

Lines 5–9 of the algorithm demonstrate the process of calculating the prior probability values of each cell by reflecting the weight values. This process is calculated for all cells in the search area; therefore, the computational complexity is O(n^2^). Line 10 of the algorithm shows the process in which the probability value of each cell is updated, and lines 11 and 12 indicate the process in which the heuristic and sensor measurement values of each cell are updated. These processes also have a computational complexity of O(n^2^) because calculations are made for all cells in the search area. Lines 13–17 show the part that verifies whether each cell contains the target, and these also have a computational complexity of O(n^2^). Because tasks such as the weighting of each cell, probability, and checking whether the target exists are repeated until the target is found, this algorithm can ultimately be concluded to have O(n^3^) computational complexity.
**Algorithm 1: Improved A-Star Algorithm**1: Initialize *P_0_, H_0_, O, S_0_*2: targetFound=0, round=13: **while** (targetFound!=1)4:  **if**(roundLimit!=1) then5:   **if**(round==1) then6:      *P_1_(i,j)=P_0_(i,j)+H_0_(i,j)+O(i,j)+S_0_(i,j)* for each *c(i,j)*7:   **else**8:    *P_t_(i,j)=P_t−1_(i,j)+H_t−1_(i,j)+S_t−1_(i,j)* for each *c(i,j)*9:   **end-if**10:    *P_t_(i,j)=Prob(c(i,j))* for each *c(i,j)*11:    *H_t_(i,j)=Heu(C_t_)*12:    *S_t_(I,j)=W(MA_t_)*13:    **if**(*P_t_*>=*Th*) then14:    targetFound=115:    **else**16:    go to next round17:    **end-if**18:    round++19:   **end-if**20: **end-while**

## 4. Simulation

Simulations were conducted to analyze the performance and effectiveness of the proposed improved A-star algorithm.

### 4.1. Simulation Environment

The simulation environment is presented in Table 2. The search area consists of 8 × 8 cells, and the target was randomly placed in one of these cells. The drone searches for a set of targets using various methods.

In this study, search probability models, such as Equations (1) and (8), were used. Determination of *α* and *β* values is important in probabilistic models [3]. Therefore, this study used the optimal values obtained through experiments for simulations, as listed in Table 3. The data of the Symington et al. [20] study were used in the simulation. To analyze the performance of the proposed method, several well-known stochastic search methods were compared.

The linear search method and high- and low-altitude collaboration search method [7] were compared with the proposed method. Linear search involves linearly searching for cells in a search area at a high or low altitude. In the search method based on high- and low-altitude collaboration, the drone first searches a wide area at a high altitude, selects the area with the highest probability, and searches at a low altitude to increase the probability of a target search. In this simulation, the performance was analyzed by applying the proposed algorithm to the following three search methods.

- Search method 1: Low-altitude linear search method (LowLinear). In this method, the drone searches linearly at low altitudes to find a target.
- Search method 2: High-altitude linear search method (HighLinear). In this method, the drone searches linearly at high altitudes to find a target.
- Search method 3: High-altitude and low-altitude collaboration search method (HighLow). In this method, high-altitude and low-altitude drones cooperate to find a target.

The time and distance required for the drone to search for a target were used to evaluate the performance of the simulation. The distance explored by the drone was obtained using the following Equation (18). In this equation, $C_{n}$ is the number of cells that the drone moves to for a search, and the tangent value is the value between base b and height h. The time required for the search was calculated using the average speed of the drone in the experimental environment, as expressed in Equation (19).

(18) $$D=C_{n}\times \sqrt{2\times (\frac{1}{\tan\theta_{hb}}\times h{)}^{2}}$$

(19) Search Time=Velocity of Drone÷Search Distance

### 4.2. Performance Analysis

**Figure 3:**
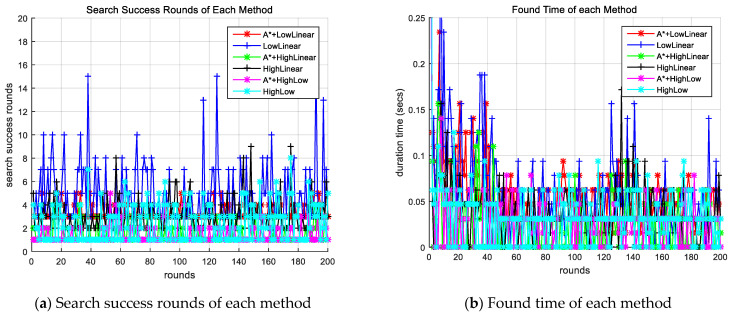
Comparison of success round and CPU time for each round of each method. A* indicates the proposed algorithm. LowLinear: low-altitude linear search method; HighLinear: high-altitude linear search method; HighLow: High-altitude and low-altitude collaboration search method.

Search methods with the proposed algorithm and other search methods search for targets through simulations, and the search results are analyzed. Figure 3a shows the search success rounds for each round of each method simulated, and Figure 3b shows the CPU time until the search success for each round of each method. The search success rounds of methods that do not apply the proposed improved A-star algorithm can be observed to be large and the CPU time long.

**Figure 4:**
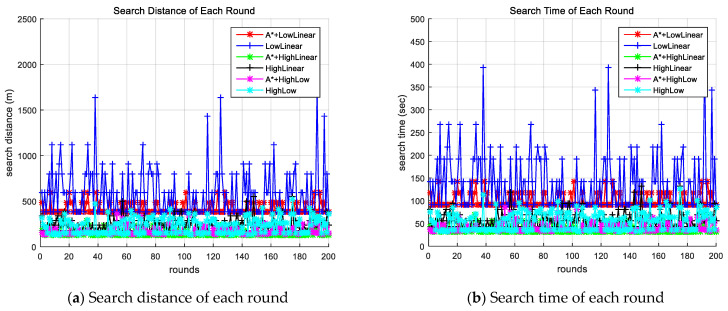
Comparison of search time and search distance for each round of each method. A* indicates the proposed algorithm.

Figure 4 shows the search distance and time for each search round of each method. Figure 4a shows the distance to successful search, and Figure 4b shows search time for each round. This result also showed that the method using the improved A-star algorithm required less search time and distance compared to the corresponding method without the proposed algorithm.

**Figure 5:**
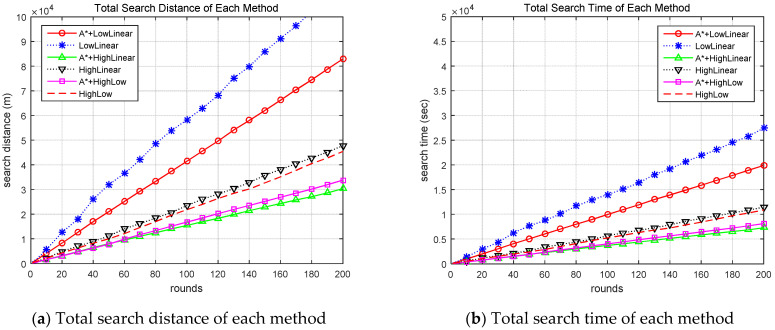
Comparison of accumulated search distance (**a**) and accumulated search time (**b**) for each method. A* indicates the proposed algorithm.

Figure 5 shows the accumulated search distances and times for each method. The LowLinear search method had the most cumulative rounds, whereas the HighLinear search and the HighLow search methods had similar cumulative rounds. However, when comparing the methods with and without improved A-star algorithm, the method using the improved A-star algorithm clearly demonstrates superior performance.

**Figure 6:**
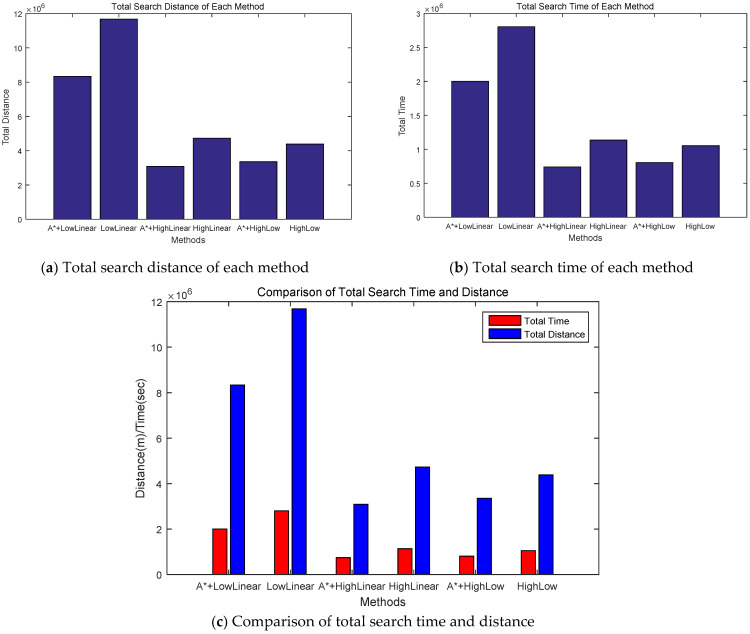
Comparison of total search distance (**a**) and total search time (**b**) for each method. (**c**) Total search distance and time for each method. A* indicates the proposed algorithm.

Figure 6a,b show the total search distance and search time for each method. The method applying the proposed improved A-star algorithm exhibited significantly better performance compared with the corresponding method without the proposed algorithm. The LowLinear search with the proposed algorithm exhibited more than 40% better performance than the LowLinear search method, whereas the HighLinear search with the proposed algorithm exhibited more than 53% better performance than the HighLinear search method. In the case of exploration using the HighLow search method, the proposed algorithm showed approximately 30% better performance than the conventional HighLow method. Figure 6c shows the total search distance and time for each method.

**Figure 7:**
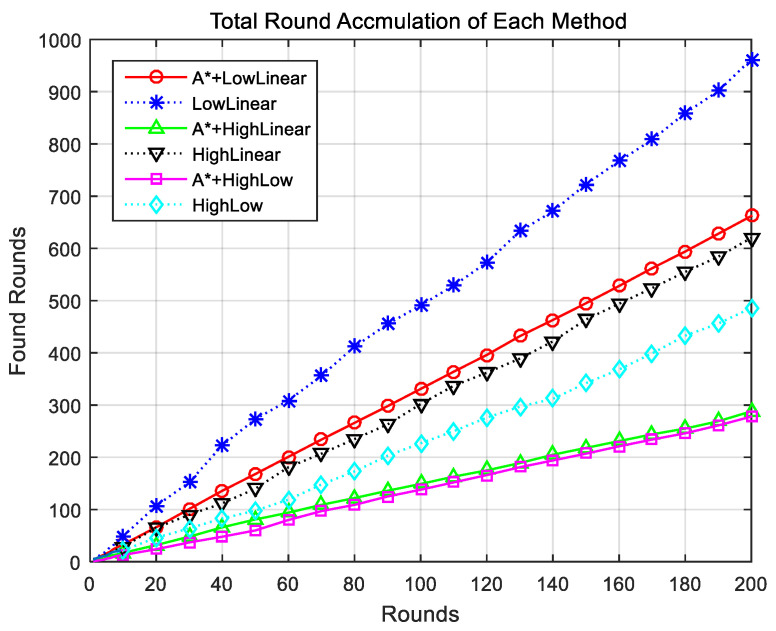
Comparison of cumulative success rounds for each method. A* indicates the proposed algorithm.

Figure 7 shows the accumulation of successful rounds for each method. The LowLinear search method showed the most cumulative rounds, whereas the HighLow method exhibited the fewest successful rounds. When comparing the method using the proposed improved A-star algorithm with the corresponding method without the proposed algorithm, the method with the proposed algorithm exhibited significantly better performance.

**Figure 8:**
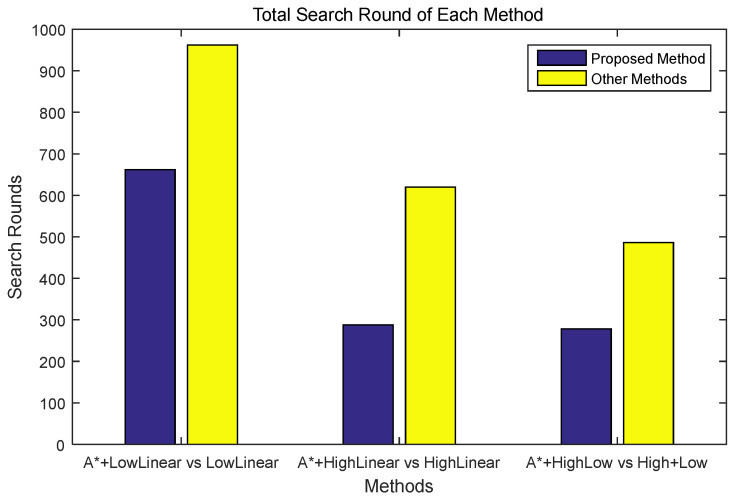
Performance comparison of each method in terms of success rounds. A* indicates the proposed algorithm.

Figure 8 shows the results of the exploration success rounds of the methods with and without the proposed algorithm. In a successful round, in the case of a low-altitude linear search, the proposed method exhibited superior performance by approximately 45%, and in the case of a high-altitude search, the proposed method exhibited superior performance by approximately 115%. In the case of high-altitude and low-altitude collaborative exploration, the proposed method exhibited superior performance of approximately 74% or more.

### 4.3. Discussion

The simulations demonstrated that when the proposed method was applied to the LowLinear search, HighLinear search, and HighLow search methods (Section 4.1), the method using the proposed improved A-star algorithm performed significantly better. Additionally, the HighLinear search and HighLow search methods were observed to perform much better than the LowLinear search. This is due to the difference between the false alarm value α and the missed detection value β used in the probability search, and the difference in travel distance because high-altitude searches a unit composed of 4 × 4 cells, whereas low-altitude searches a unit composed of 2 × 2 cells.

As shown in the search results, when comparing the high-altitude search with the high-altitude and low-altitude collaboration search, the search round shows that the high-altitude and low-altitude collaboration search exhibit superior performance owing to the collaboration algorithm; however, the search distance and time are different. In this case, the performance was slightly lower because the search distance at low altitudes increased. However, in all three search methods described in Section 4.1, the method applying the proposed improved A-star algorithm can be observed to perform much better than the conventional method.

Therefore, the simulations demonstrated that the improved A-star algorithm proposed in Section 4.1 is effective when applied to the three types of existing probabilistic search algorithms mentioned. In other words, comparing the probabilistic search algorithm with the improved A-star algorithm with the one without the improved A-star algorithm, the application of the improved A-star algorithm proved to be superior in terms of search time, search distance, and search success rate in the probabilistic search algorithm. This shows that the proposed algorithm can be used in actual navigation of drones.

The dataset used in the simulation was obtained from practical experiments detecting ground targets [5,7]. Because actual experimental data for air pollution exploration were not available, this dataset for ground target detection was utilized. However, applying various experimental data at different altitudes, aside from those presented in Table 3, yielded similar results. Therefore, the proposed algorithm is anticipated to be effective across a wide range of real-world experimental data.

In this simulation, the drone repeatedly searches the search space and checks whether the probability of each cell reaches a value above the threshold, increasing the probability of finding air pollution. Therefore, based on the probabilistic model used in this study, it is difficult for the drone’s path to proceed while maintaining a nearly consistent direction toward the target. Therefore, an improved A-star algorithm was proposed to reduce the search distance, which is a disadvantage of the stochastic model, and to obtain information about the direction of search.

## 5. Conclusions

In this study, an improved adaptive A-star algorithm that can be used in the probabilistic search of drones to identify air pollution sources in urban areas was proposed. In particular, to increase the probability of target discovery in the proposed algorithm, a weight value $H\left(n\right)$ based on the expected distance to the target, weight value S(n) based on the measurement value of the target search sensor, and weight value $O\left(n\right)$ based on the initial presence of obstacles were used. These values are added to the previous search probability values to calculate a new search probability value. Through several simulations, high-altitude linear search, low-altitude linear search, and high-altitude and low-altitude collaboration search methods were selected, and the conventional search algorithm was compared with the proposed improved A-star algorithm. In terms of search distance and search time, the proposed algorithm exhibited significantly better performance than the conventional algorithm for all three methods, ranging from approximately 30% to approximately 53%. In addition, in terms of search success rounds, the method using the proposed algorithm exhibited superior performance of approximately 45% and up to 115% compared with the method with the conventional algorithm.

The method proposed in this study has the following limitations. First, the stochastic search method repeatedly searches the search space and increases the probability of success in the search; therefore, the distance and time of the search are likely to be long. This is a common problem with stochastic search methods. However, owing to the characteristics of air pollution, repeated searches may be necessary. Second, the proposed improved A-star algorithm was applied in a two-dimensional space in the simulation. However, the proposed algorithm will be easily extended to three-dimensional space. Even if a space where air pollution exceeds the threshold is found, air pollution can move depending on climate changes such as wind and rain. Therefore, air pollution detection drones may need to continuously conduct repetitive searches. Therefore, additional simulation work needs to be performed in the future to expand the proposed model to three-dimensional space. Furthermore, additional research is needed to increase search time and performance by applying the proposed improved A-star model to other possible probabilistic search models.

Our study has the following significance. First, this study extends the traditional A-star algorithm to a drone-based urban air pollution search algorithm. The A-star algorithm was improved to be suitable for air pollution exploration by considering the fluid and invisible nature of air pollution. Second, the proposed improved A-star algorithm was applied to a probabilistic search model for air pollution search. By applying the proposed algorithm to the existing probabilistic search model, the performance of probabilistic search was improved. Third, an algorithm that can be used for practical air pollution exploration using drones is proposed. There are a few algorithms for detecting the presence of air pollutants with fluid properties. Therefore, the results of this study can serve as a basis for future air pollution detection and environmental research.

## Figures and Tables

**Table 2:** Simulation environment.

Category	Value
Search area	8 × 8 cells
Speed	15 km/h (4.1666667 m/s)
Altitudes	High altitude: 20 m (1 unit = 4 × 4 cells) Low altitude: 10 m (1 unit = 2 × 2 cells)
Threshold	0.95
Length of a side	7.592 m
Targets	1 (random)
UAVs	1
Limitations on navigation rounds	200

**Table 3:** Experimental values of *α* and *β*.

Altitude	Dataset 1 [5]		Dataset 2 [7]	
	*α*	*β*	*α*	*β*
10 m	0.06286	0.20000	0.028369	0.000000
20 m	0.00130	0.34593	0.001110	0.046745

## Data Availability

Data are contained within the article.
